# Nutrient availability increases photosynthetic capacity without altering the cost of resource use for photosynthesis

**DOI:** 10.1093/aobpla/plaf061

**Published:** 2025-10-22

**Authors:** Jan A Lankhorst, Hugo J de Boer, Dorian C Behling, Paul L Drake, Evan A Perkowski, Karin T Rebel

**Affiliations:** Environmental Sciences, Copernicus Institute of Sustainable Development, Utrecht University, Princetonlaan 8a, 3584 CB Utrecht, The Netherlands; Environmental Sciences, Copernicus Institute of Sustainable Development, Utrecht University, Princetonlaan 8a, 3584 CB Utrecht, The Netherlands; Wageningen University, Droevendaalsesteeg 4, 6708 PB, Wageningen, The Netherlands; School of Biological Sciences, University of Western Australia, 35 Stirling Highway, 6009 Perth, WA, Australia; Department of Biological Sciences, Texas Tech University, 2500 Broadway, 79409 Lubbock, TX, United States; Environmental Sciences, Copernicus Institute of Sustainable Development, Utrecht University, Princetonlaan 8a, 3584 CB Utrecht, The Netherlands; Plants, Ecosystems & Climate

**Keywords:** least-cost optimality, nutrient availability, photosynthetic capacity, plant physiology, nitrogen uptake, *Solanum dulcamara*, *Holcus lanatus*

## Abstract

Eco-evolutionary optimality (EEO) theory predicts that plants maximize resource investment in photosynthetic capacity at the lowest costs of acquiring and using such resources. However, current EEO-based models predict photosynthetic capacity based on climate alone, and omit costs for resource acquisition. To explore the link between leaf-level optimality and plant-level nitrogen acquisition costs across different soil environments, we grew two commonly co-occurring species in a greenhouse under three nutrient fertilization levels in sand and two natural soils with matching nutrient availability to the fertilization levels in sand. At the end of the experiment, we measured the maximum rate of Rubisco carboxylation (*V*_cmax_), δ¹³C-derived leaf-to-air CO_2_ partial pressure ratio (*c_i_*/*c_a_*), and structural carbon costs for nitrogen acquisition. Increasing nutrient availability increased *V*_cmax_ (*P* < .001) and decreased carbon costs for nitrogen acquisition (*P* < .001), similarly in sand and natural soils (*P* > .1 for both). Yet, the leaf *c_i_*/*c_a_* remained unchanged across treatments in sand (*P* = .426) and natural soils (*P* = .499), consistent with the current EEO-models assumption of climate-dependent optimality. These findings support the general principle that nutrient scarcity increases acquisition costs, while also highlighting a gap in current model formulations that neglect nutrient effects on photosynthetic acclimation.

## Teaser

Recent developments in land surface modelling allow the prediction of photosynthetic capacity from climate following least-cost optimality principles. Yet, soil nutrient availability introduces unexplained variation. This study shows nutrient availability increases photosynthetic capacity without altering the cost of resource use for photosynthesis.

## Introduction

Accurate representation of the factors that influence photosynthetic capacity is crucial for capturing ecosystem responses to future climatic conditions (Prentice et al. 2015). Current land surface models differ in their representations of the hydrological, carbon, and nutrient cycles, leading to diverging predictions of the land carbon sink (Prentice et al. 2015, Harrison et al. 2021, Stocker et al. 2025). Studies often show diverging responses of leaf-level photosynthesis to nitrogen and phosphorus availability, with some suggesting positive impacts of increasing nutrient availability on photosynthetic capacity and others showing null or negative responses (Liang et al. 2020, Stocker et al. 2025). This lack of consistent plant responses to nutrient availability suggests a need for further experimentation to understand the mechanisms that link photosynthetic responses to nutrient availability.

Eco-evolutionary optimality (EEO) theory offers a promising approach for a quantitative representation of vegetation dynamics by mechanistically linking plant physiological traits to resource demand and acquisition (Prentice et al. 2011, Harrison et al. 2021 , Wang et al. 2023). EEO theory uses first principles of photosynthetic least-cost theory, asserting that plants allocate resources—specifically nutrients and water—towards photosynthetic enzymes and structures to maintain a transpiration stream as a strategy for maximizing light-use efficiency and carbon gain at the lowest cost of maintaining these processes (Wright et al. 2003, Prentice et al. 2014). The central premise is that both resources are substitutable, meaning that leaves in dry areas can compensate for reduced stomatal conductance due to more costly water acquisition and use by increasing investment in photosynthetic capacity and vice versa in wetter, more fertile areas (Wright et al. 2003, Prentice et al. 2014, Perkowski et al. 2025b). The theory posits that plants optimize the coordination of key limiting steps of photosynthetic reactions, such that net photosynthesis rates are co-limited by the maximum rate of Ribulose-1,5-bisphosphate (RuBP) carboxylase/oxygenase (Rubisco, *V*_cmax_) and the maximum rate of electron transport for RuBP regeneration (*J*_max_) (Chen et al. 1993, Maire et al. 2012). At the leaf-level, EEO theory allows for the prediction of optimal trait combinations focusing on the tradeoff between carbon gain and water loss (Wright et al. 2003, Prentice et al. 2014, Wang et al. 2017, Smith et al. 2019, Stocker et al. 2020), through the close coordination between *V*_cmax_ and *J*_max_, as well as the ratio of leaf intercellular CO_2_ partial pressure to ambient CO_2_ partial pressure (*c_i_*/*c_a_*). EEO theory states that plants optimize their *c_i_*/*c_a_* ratio, termed *χ*, by altering investment in *V*_cmax_ and stomatal conductance, allowing individual leaves to maximize carbon gain by minimizing the summed costs of acquiring and using nutrients and water (Smith et al. 2019, Perkowski et al. 2025b).

In current EEO theoretical frameworks, the costs of nitrogen acquisition are assumed to be constant across nitrogen availability (Prentice et al. 2014, Wang et al. 2017, Stocker et al. 2020). While recent findings show that soil nutrient availability influences the leaf-level costs for maintaining carboxylation and transpiration (Paillassa et al. 2020, Westerband et al. 2023, Cheaib et al. 2025), these patterns have not been incorporated into EEO theory yet. The current static formulation is based on a global assessment of leaf-level relative costs using leaf carbon isotope data (Wang et al. 2017, Stocker et al. 2020) and partially based on the assumption that plant-level investments in root infrastructure equally benefit uptake of water and nitrogen (Prentice et al. 2014). However, previous work highlights that costs of nitrogen acquisition vary in species with different uptake strategies and across nitrogen availability gradients (Shi et al. 2016, Allen et al. 2020, Perkowski et al. 2021, 2024, 2025a, Waring et al. 2023), which may benefit the uptake of nitrogen more than that of water, changing the ratio of resource-acquisition costs. When soil nitrogen is abundant, costs of nitrogen acquisition are generally decreased because uptake of inorganic nitrogen can occur passively through diffusion and convection via root water uptake (Fisher et al. 2010). On the other hand, when nitrogen is less abundant (e.g. due to increased nitrogen stored in organic matter), costs to acquire nitrogen generally increase because plants must invest more in root tissue construction and maintenance for either similar or reduced nutrient returns on root investment (Bergmann et al. 2020, Perkowski et al. 2021). As an alternative strategy to overcome the increased costs of acquiring nitrogen, plants can form symbiotic relationships with microorganisms—such as nitrogen-fixing bacteria, arbuscular mycorrhizae, or ectomycorrhizae—which allow them to access otherwise unavailable nitrogen sources in nitrogen-poor systems. However, this comes with additional carbon costs to maintain these symbioses (Rastetter et al. 2001, Shi et al. 2016, Terrer et al. 2018).

Similarly, phosphorus uptake—whether direct or microbially mediated (Raven et al. 2018)—incurs variable costs and significantly influences plant productivity and photosynthesis (Vitousek et al. 2010, Peng et al. 2021, Ellsworth et al. 2022, Lu et al. 2022). Phosphorus uptake schemes have only recently been incorporated into a small number of global models with varying effects (Jiang et al. 2024), likely due to a lack of understanding regarding the mechanisms governing plant phosphorus uptake and downstream effects of phosphorus availability on investment in photosynthetic processes (Ellsworth et al. 2022).

The existence of differences in nitrogen and phosphorus uptake mechanisms due to changes in soil nutrient availability suggests that carbon costs for nutrient acquisition may not be constant and, more importantly, may not be proportional to the cost of water acquisition, as is currently assumed by EEO-theory (Allen et al. 2020, Paillassa et al. 2020, Perkowski et al. 2021, 2024). Therefore, manipulation experiments that seek to address the mechanism governing the link between leaf-level optimality and costs of nutrient acquisition are necessary in order to better understand how these patterns should be implemented in future EEO frameworks.

We investigated the current EEO assumption that plant-level investment in root biomass equally benefits direct nutrient- and water uptake to maintain the leaf-level tradeoff between carboxylation capacity and maintaining a transpiration stream. We tested the following hypotheses:

- Increasing plant-available nutrients will decrease the carbon costs to acquire nitrogen, driven by a larger increase in whole-plant nitrogen biomass than in root carbon biomass.
- Increasing plant-available nutrients will decrease leaf-level *χ* values by decreasing the leaf-level costs for maintaining carboxylation compared to transpiration.

To test these hypotheses, we grew two commonly coexisting species in a gradient of three increasing inorganic nutrient (nitrogen and phosphorus) treatments, and in two different native soils with known quantities of inorganic nitrogen and phosphorus while maintaining nonlimiting water availability. Thus, changes in the cost for acquiring and using nutrients relative to water are expected to be entirely driven by a change in the cost for acquiring nutrients, following previous work (Perkowski et al. 2021, 2024, 2025a). Plants were measured for their photosynthetic capacity, isotope-derived *χ* values, and plant-level costs for nitrogen acquisition.

## Materials and methods

### Experimental setup

#### Plant cultivation

The nutrient addition experiments were conducted in 2022 and 2023, while the natural soils experiments were conducted only in 2023. All experiments were conducted in the greenhouse of the botanical gardens of Utrecht University. Plants were grown inside a naturally lit compartment, without temperature control during the months May–August. This resulted in a naturally varying daylight and temperature, averaging 23.8°C in 2022 and 24.8°C in 2023 (Supplementary Table SI1). Seeds of *Solanum dulcamara* L. and *Holcus lanatus* L. provided by Cruydt Hoeck (Nijeberkoop, NL) were germinated in wet, sterilized sand (see: Nutrient treatments). Two weeks after germination, seedlings were selected based on similar growth stage and individually transplanted to their treatment pots. All plants were grown in 3-L pots (CEP, 19.2 cm × 15 cm), with deep trays positioned beneath the pots to prevent excessive nutrient loss as leachate. Field capacity was determined gravimetrically by weighing pots with dry substrate and again when leachate was first observed in the trays; watering during the experiment was adjusted to reach this weight without causing excessive leachate. Growing seasons differed slightly between years, with the 2022 experiment lasting 9 weeks from the beginning of May until mid-July, and the 2023 experiment lasting 10 weeks from the end of May until mid-August. Climatic variables were measured using greenhouse loggers, and differences in growing conditions are summarized in Supplementary Table SI1 and in Supplementary Figure SI1. All pots were randomized weekly following a completely randomized design to account for possible artifacts associated with heterogeneity in growth conditions within the greenhouse area. As climatic environments were assumed to be similar for all plants within the same year, demand for nitrogen was assumed to be similar between all treatments, making supply of nutrients the only growth factor that was manipulated across the experiment.

#### Nutrient treatments

A total of 104 pots were used over the course of the experiment (60 in 2022, 44 in 2023). Each pot was filled with autoclaved coarse river sand without organic matter. The sand was autoclaved at 121°C for 45 min. Three nitrogen (N) and phosphorus (P) treatments were established: low (26 mg N, 1.75 mg P), medium (36 mg N, 2.4 mg P), and high (78 mg N, 5.25 mg P). For the 2022 experiment, 20 pots were used for the low treatment, 20 pots for the medium treatment, and 20 pots for the high treatment. In 2023, 16 pots were used for the low treatment, 14 pots for the medium treatment, and 14 pots for the high treatment. Nutrient treatments were based on the average N and P values observed in the natural soils, with the respective nutrient solutions derived from the modified Hoaglands solution of Wang et al. (2021b), with an N:P ratio of 15 for all treatments (a detailed recipe is provided in Supplementary Table SI4). All other elements were supplied in the same amounts in all treatments, with only Cl varying. Consequently, nutrient availability consists of both N and P, in the same ratio and therefore covarying in the same magnitude. By keeping all elements the same between treatments, NH_4_^+^ to NO_3_^−^ ratios differed slightly between treatments. Weekly nutrient additions consisted of 150 mL of the nutrient solutions during the growth seasons.

#### Natural soils

A total of 36 pots were filled with either of two types of natural soil (14 pots for Microp soil, 22 pots for Reijerscamp soil). For all pots, 3.9 kg of air-dried substrate was used to fill each pot. The need to standardize the amount of soil in each pot constrained the sample size due to the limited availability of Microp soil. This soil type was provided by the Dutch Gravitation program, Microbial Imprinting for Crop Resilience (MiCrop). A detailed analysis of the soil was provided by Eurofins Agro, which categorized the soil as sandy soil, with silt and clay fractions both below 10%. Plant available N was measured at 8.5 mg/kg, and plant available P at 0.5 mg/kg. With 3.9 kg soil per pot, this resulted in 33.15 mg N and 1.95 mg P readily available to each plant, assuming that the total pot volume could be exploited by roots. The second soil type was obtained from a collection campaign in Reijerscamp, a nature reserve in the Netherlands (52.01° N, 5.781° W). Soil was sourced from a depth of 5–20 cm from the surface, after removing the topsoils, including small grasses that occupied this area. This reserve was selected as both species used in the study are endemic, and the soil in the Reijerscamp nature reserve was classified as sandy with very low silt and loam fractions, consistent with the other substrates used in this experiment [BIS-Nederland, Version 7, Wageningen Environmental Research (Alterra)]. The Reijerscamp soil was collected at the beginning of March 2023 and allowed to air-dry before sieving.

All soils were compared to the existing analysis of the Microp soil using two KCl extracts of +−200 g well-mixed soil on the Discrete Analyser (Gallery, Thermo Scientific™) for [NH_4_^+^] and [NO_3_^−^] and colorimetric analysis on the same soil quantities extracted with NaHCO_3_ for [PO_4_^−^] (Table 1) (Murphy and Riley 1962). Values from these analyses for the Microp soil differed only slightly from the UNIFARM analyses and were used for the statistical analyses. The Reijerscamp soil had over 1.5 times the amount of available N compared to the Microp soil, with both increased [NH_4_^+^] and [NO_3_^−^].

**Table 1.** Comparison and composition of nutrients added to the sand experiment and discrete analyser results for soil available nutrients in the natural soils experiment, scaled to whole-pot concentration.

Method	Nutrients added to sand			Discrete analyser (DA) or colorimetric analysis (CA)	
	Sand low	Sand medium	Sand high	Microp	Reijerscamp
Total N (mg/pot)	26	36	78	38.25 ± 1.88 (DA)	58.8 ± 2.72 (DA)
NH_4_^+^:NO_3_^−^	1:2.18	1:2.01	1:1.57	1:1.53	1:1.43
Total P (mg/pot)	1.75	2.4	5.25	3.38 ± 0.7 (CA)	4.96 ± 0.5 (CA)

Measured values in mean ± SD.

### Measured traits

#### Leaf gas exchange and leaf area

In the 2 weeks prior to destructive sampling, at least five distinct plants per species per treatment were measured for photosynthetic capacity using a LiCOR 6400 XT (Li-COR Biosciences, Lincoln, NE, USA) with a 3 cm × 2 cm RB light source (6400-02B LED Light Source). The most recently fully expanded mature leaf was chosen for *S. dulcamara*, and the tiller with the highest number of healthy, fully expanded leaves for *H. lanatus*. A modified protocol was used based on Evans and Santiago (2014) to construct steady-state relationships between photosynthetic rate (*A*, µmol m^−2^ s^−1^) and intercellular CO_2_ (Ci, µmol mol^−1^), termed *A*/Ci curves, and relationships between *A* and photosynthetically active radiation (µmol m^−2^ s^−1^), termed *A*/*Q* curves. *A*/Ci curves were generated under greenhouse saturated light conditions obtained from the *A*/*Q* curves (1500 μmol m^−2 ^s^−1^), a relative humidity between 40% and 60% to allow for sufficient stomatal conductance, and constant chamber temperature (25°C) using the following steady-state reference CO_2_ concentration (ppm) sequence: 400, 200, 50, 100, 400, 600, 900, 1500, 400. The maximum rates of Rubisco carboxylation (*V*_cmax_; μmol m^−2^ s^−1^) and electron transport for RuBP regeneration (*J*_max_; μmol m^−2^ s^−1^) were estimated from the *A*/Ci curve data and standardized to 25°C using the ‘fitaci’ function from the R-package ‘plantecophys’ (Duursma 2015). As estimates of *V*_cmax_ and *J*_max_ were derived using Ci rather than mesophyll [CO_2_], the estimated values for *V*_cmax_ are likely to be slight underestimates of the true values, but we expect that this method does not directionally affect the treatment responses.

Leaves measured for photosynthetic capacity were scanned and their projected surface area measured using ImageJ (Schneider et al. 2012). With respect to elemental analyses (see further details below), these specific leaves were analysed separately from the bulk of each plant’s leaf biomass. Dry weight was determined after oven-drying leaves for at least 48 h at 60°C.

#### Plant biomass and elemental analysis

After gas exchange measurements, experimental plants were destructively sampled for biomass, LMA (from leaf scans above), carbon and nitrogen content, and stable carbon isotope ratio (δ^13^C). For dry mass, plants were divided into roots, leaves, and stems, where ‘stems’ for *H. lanatus* were identified as leaf sheaths, not blades. Dry weight was measured by drying leaves, stems, and washed roots separately at 60°C for at least 48 h and weighing separately on an analytical balance. Plants in year 2 grew 1 week longer than in year 1, resulting in greater total biomass in 2023 compared to 2022. In 2022, with the exception of the *H. lanatus* in the highest nutrient treatment, all plants of both species had lower average dry biomass/pot volume ratios than the 1:1 (g L^−1^) ratio recommended by Poorter et al. (2012a, 2012b) to minimize the likelihood of pot volume-induced growth limitation. In 2023, this ratio was exceeded, but all plants showed similar response patterns as in 2022, making pot size-induced growth limitation unlikely.

Dried material was ground using a Retsch MM200 ball-mill grinder (Verder Scientific, Inc., Newtown, PA, USA) with tungsten balls. Carbon and nitrogen content (g g^−1^) was measured using a Thermo Scientific™ Flash IRMS™ Elemental Analyser (Waltham, MA, USA), with roots, stems, and leaves analysed separately. We scaled these values to total leaf, stem, and root carbon and nitrogen biomass (g) by multiplying carbon and nitrogen content with the dry biomass of each organ type. Whole-plant nitrogen biomass (gN) was calculated as the sum of total leaf (gN), stem (gN), and root (gN) nitrogen biomass. Stable carbon isotope content (δ^13^C_leaf_, ‰, relative to VPDB) was measured using the Thermo Scientific™ Isotope Ratio Mass Spectrometer and used to calculate leaf-lifespan averaged *c_i_*/*c_a_* values (Farquhar et al. 1989, Scher et al. 2022):


(1) $$\Delta^{13}C_{leaf}=\frac{\delta^{13}C_{air\;}-\;\delta^{13}C_{leaf}}{1\;+\;(\delta^{13}C_{leaf}/1000)}$$

(2) $$\chi_{iso}=\frac{\Delta^{13}C_{leaf}\;-\;a}{b\;-\;a}$$

where Δ^13^C is the carbon isotope discrimination between plant tissue and the surrounding atmospheric CO_2_, calculated from leaf δ^13^C measures and air δ^13^C values as derived in Feng (1999). The isotope-derived leaf-lifespan *c_i_*/*c_a_* ratio, termed *χ*_iso_, is calculated using this leaf-tissue discrimination and ‘*a*’ the fractionation during diffusion into the stomata (assumed to be constant 4.4‰; Farquhar et al. 1989) and ‘*b*’ the fractionation during carbon fixation due to RuBisCO preferentially fixing ^12^C (assumed to be constant at 27‰; Farquhar et al. 1989). Using *χ*_iso_ provides a measurement integrating stomatal conductance and photosynthesis across the lifespan of the leaf, and thus reduces the sub-hourly variability in stomatal conductance expected when using gas-exchange measurements from plants measured over the course of a single day. Integrating stomatal conductance over leaf lifespan also aligns more closely with whole-plant time scales of response.

We quantified the carbon costs for nitrogen acquisition as root structural carbon per unit whole-plant nitrogen biomass (gC gN^−1^) following the formulation presented in Perkowski et al. (2021). This calculation uses root carbon biomass as an estimation of carbon allocated towards nitrogen acquisition at the whole plant scale. While this carbon cost does not directly capture nonstructural carbon costs through respirational carbon, carbon transferred to mycorrhizae, exudated through root exudation, or lost through root turnover, those costs are assumed to be proportional to standing root carbon biomass (Hobbie 2006, Perkowski et al. 2021). As we quantified root structural carbon costs for nitrogen acquisition destructively at the end of the experiment, our approximations underestimated total plant carbon cost for nitrogen acquisition. As we scaled both the nitrogen and phosphorus addition in tandem in our treatments, we used only the carbon costs for nitrogen uptake as a measure of plant-level investment in nutrient acquisition.

### Statistical analysis

We explored the effects of nutrient availability in sand and in natural soils on physiological and physical plant responses using separate linear models for each response variable. For the nutrient addition experiment (sand pots, two experimental years), we fitted linear models with nutrient treatment, species, and their interaction, and year as fixed effects. Additional models, including interaction terms between year and species, and between year and treatment, were also tested and are presented in the Supporting Information (Supplementary Table SI2).

For the comparison between nutrient addition in sand and nutrient availability in natural soils, we used linear models with total nitrogen amount (mg N per pot, either added or measured), species, their interaction, and substrate (sand vs. natural soil) as fixed effects. Because phosphorus content varied slightly between natural soils and the nutrient addition experiment, we included additional models, including phosphorus instead of nitrogen, in the Supporting Information (Supplementary Table SI3). Only data from the nutrient addition experiment in the same year were used to prevent a growth-season effect.

The linear models residuals were tested for normality using Shapiro–Wilk tests (*P* > .05). All models except the model for *N*_area_ satisfied the assumptions of normality (*P* = .046). After log-transforming, *N*_area_ model residuals satisfied the assumption of normality.

All linear models were created using the ‘lm’ function in R. The significance of fixed effects was assessed using Type II ANOVA via the ‘Anova’ function from the car package (Fox and Weisberg 2019). *Post hoc* comparisons were conducted using the emmeans package, with Tukey HSD-adjusted *P*-values for multiple testing (Lenth 2024). All analyses and plots were performed in R version 4.3.1 (R Core Team 2023).

## Results

### Effects of nutrient amendments on plant resource allocation

**Figure 1:**
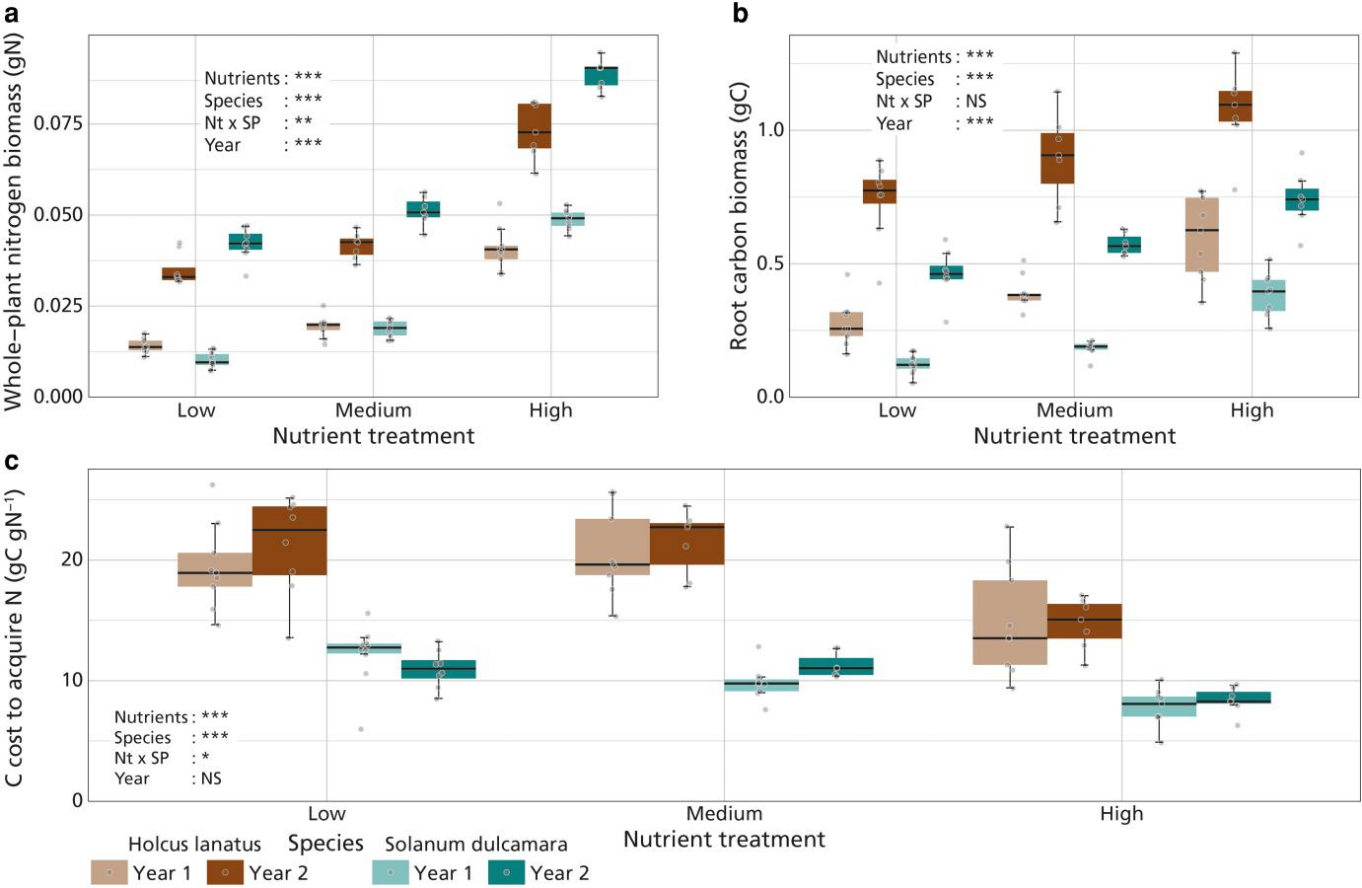
. Effects of nutrient treatment (*x*-axis) on a) whole plant nitrogen biomass, b) total root carbon biomass, and c) carbon cost to acquire nitrogen (gC gN^−1^). *H. lanatus* is represented in the left two boxes per nutrient treatment (brown colours), *S. dulcamara* is represented in the right two boxes per nutrient treatment (green colours). The different years are represented by the different shades per species. Boxplots represent the sample median, and the first and third quartiles and 1.5 times the interquartile range. Points represent the individual measurements and are jittered for visibility. Significance from ANOVA analysis is represented by: *** = *P* < .001; ** = *P* < .01; * = *P* < .05, NS = *P* > .05 (Table 2).

Whole-plant nitrogen biomass increased (gN) with nutrient fertilization (Fig. 1a, *P* < .001, Table 2), with a significantly greater nitrogen biomass in *S. dulcamara* compared to *H. lanatus* (*P* < .001, Table 2). Additionally, a significant interaction between nutrients and species was observed (*P* = .006, Table 2), with *S. dulcamara* showing a stronger increase in whole-plant nitrogen biomass to nutrient addition than *H. lanatus*. Whole-plant nitrogen biomass was significantly greater in the second year (*P* < .001, Table 2). Root carbon biomass (gC) also increased with nutrient addition (Fig. 1b, *P* < .001, Table 2), with significantly greater root carbon biomass in *S. dulcamara* (*P* < .001, Table 2) and in the second year (*P* < .001, Table 2), but no interaction between nutrient addition and species (*P* = .469, Table 2). Carbon costs for nitrogen acquisition showed a significant decrease with increasing nutrient fertilization (Fig. 1c, *P* < .001, Table 2), with *H. lanatus* having a significantly greater cost compared to *S. dulcamara* (Fig. 1c, *P* < .001, Table 2) but no significant difference between the 2 years (*P* = .351, Table 2). The interaction between nutrient treatments and species (*P* = .028, Table 2) indicated that, while both species showed a decrease in carbon cost for nitrogen acquisition with increasing nutrient availability, a stronger reduction was found in *H. lanatus* than in *S. dulcamara*. Additional models exploring species by year and treatment by year interactions revealed inter-annual variation in species responses (root carbon biomass, *P* = .006, and whole-plant nitrogen biomass, *P* < .001, Supplementary Table SI2) and in treatment effects (whole-plant nitrogen biomass, Supplementary Table SI2), but no significant interactions for the carbon cost to acquire nitrogen.

**Table 2.** ANOVA results exploring the effect of nutrient treatment, species, year, and their interaction on root carbon biomass, whole-plant nitrogen biomass, and carbon costs for nitrogen acquisition.

Independent variable	Whole-plant nitrogen biomass		Root carbon biomass		Carbon cost for nitrogen acquisition	
	*F*-statistic	*P*-value	*F*-statistic	*P*-value	*F*-statistic	*P*-value
Nutrients	470.4	**<**.**001**	64.7	**<**.**001**	27.5	**<**.**001**
Species	25.5	**<**.**001**	129.0	**<**.**001**	242.7	**<**.**001**
Year	806.3	**<**.**001**	373.2	**<**.**001**	0.9	.351
Nutrients:species	8.0	.**006**	0.8	.469	3.7	.**028**
Adjusted *R*^2^	0.947		0.865		0.753	

*Only data of the nutrient addition experiment is used. Significance determined using Type II *F*-tests (*α* = 0.05). *P*-values < .05 are indicated in bold and *P*-values between .05 and .100 are in italics.

These results suggest that nutrient addition reduced the carbon cost for acquiring nitrogen, primarily through a greater increase in whole plant nitrogen biomass relative to root carbon biomass. Although we observed significant differences between years in whole-plant nitrogen biomass and in root carbon biomass, there was no significant difference in carbon cost to acquire nitrogen between years.

### Effects of nutrient amendments on photosynthetic traits

**Figure 2.**
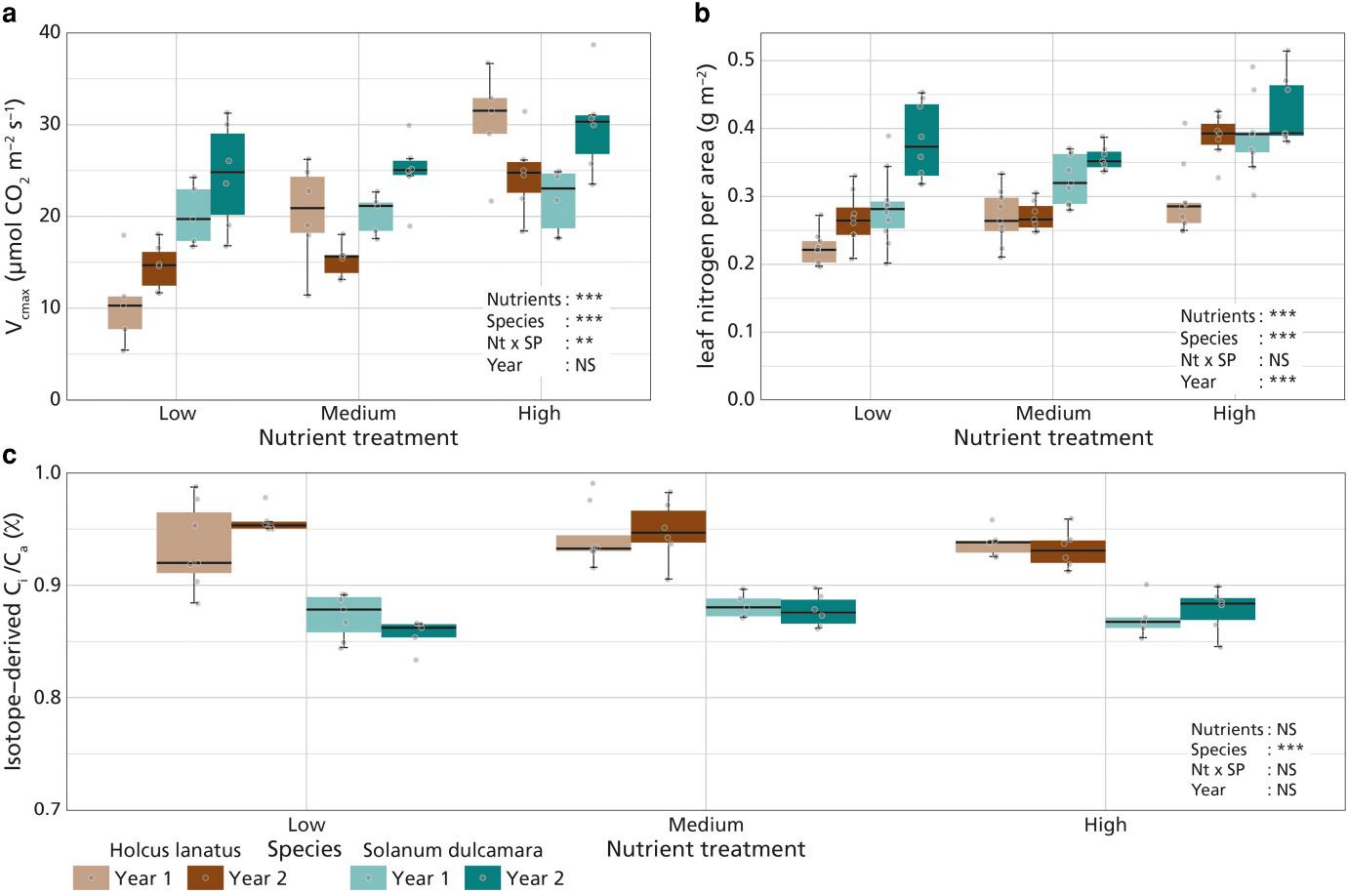
Effects of nutrient treatment (*x*-axis) on a) maximum carboxylation capacity (*V*_cmax_, µmol m^−2^ s^−1^), b) leaf nitrogen per area (g m^−2^), and c) isotope-derived *c_i_*/*c_a_* (*χ*_iso_) values. *H. lanatus* is represented in the left two boxes per nutrient treatment (brown colours), *S. dulcamara* is represented in the right two boxes per nutrient treatment (green colours). The different years are represented by the different shades per species. Boxplots represent the sample median, and the first and third quartiles and 1.5 times the interquartile range. Points represent the individual measurements and are jittered for visibility. Significance from ANOVA analysis is represented by: *** = *P* < .001; ** = *P* < .01; * = *P* < .05, NS = *P* > .05 (Table 3).

The maximum rate of carboxylation (*V*_cmax_, µmol m^−2^ s^−1^) significantly increased with increasing nutrient addition (Fig. 2a, *P* < .001, Table 3). A significant effect of species was present (*P* < .001, Table 3), with *S. dulcamara* exhibiting greater carboxylation capacity than *H. lanatus*. The interaction between nutrient treatment and species (*P* = .002, Table 3) indicated that, while *V*_cmax_ in both species increased with increasing nutrient availability, *H. lanatus* experienced a stronger response in *V*_cmax_ to nutrient availability than *S. dulcamara*. Experiment year did not significantly affect *V*_cmax_ (*P* = .164, Table 3). Leaf nitrogen per unit leaf area increased with increasing nutrient fertilization (Fig. 2b, *P* < .001, Table 3), but also significantly differed between years (*P* < .001, Table 3) and species (*P* < .001, Table 3), with greater leaf nitrogen content observed in *S. dulcamara* than *H. lanatus*. No interaction effect was found between nutrient treatment and species (*P* = .504, Table 3), indicating that both species reacted similarly to nutrient addition. Nutrient addition and year had no significant effect on isotope-derived *c_i_*/*c_a_* (*χ*_iso_) values (Fig. 2c, *P* = .426, *P* = .726, respectively, Table 3), which only significantly differed between species (*P* < .001, Table 3), with *H. lanatus* having a greater *χ*_iso_ compared to *S. dulcamara*. Additional models exploring interactions between species and year, and between treatment and year, indicated inter-annual variation in species responses for *V*_cmax_ (*P* < .001, Supplementary Table SI2) and in treatment effects for *N*_area_ (*P* = .028, Supplementary Table SI2), but no significant interactions were found for *χ*_iso_.

**Table 3.** ANOVA results exploring the effect of nutrient treatment, species, year, and their interaction on *V*_cmax_, log transformed leaf N per area, and isotope derived *χ*.

Independent variable	*V*_cmax_		Leaf*N*_area_		χ_iso_	
	F-statistic	*P*-value	*F*-statistic	*P*-value	*F*-statistic	*P*-value
Nutrients	19.1	**<**.**001**	33.0	**<**.**001**	0.9	.426
Species	13.7	**<**.**001**	70.4	**<**.**001**	182.5	**<**.**001**
Year	2.0	.164	33.4	**<**.**001**	0.1	.726
Nutrients:species	7.1	.**002**	0.7	.504	1.2	.312
Adjusted *R*²	0.486		0.690		0.717	

*Only data of the nutrient addition experiment is used. Significance determined using Type II *F*-tests (*α* = 0.05). *P*-values < .05 are indicated in bold and *P*-values between .05 and .100 are in italics.

### Substrate effects on measured traits

**Figure 3.**
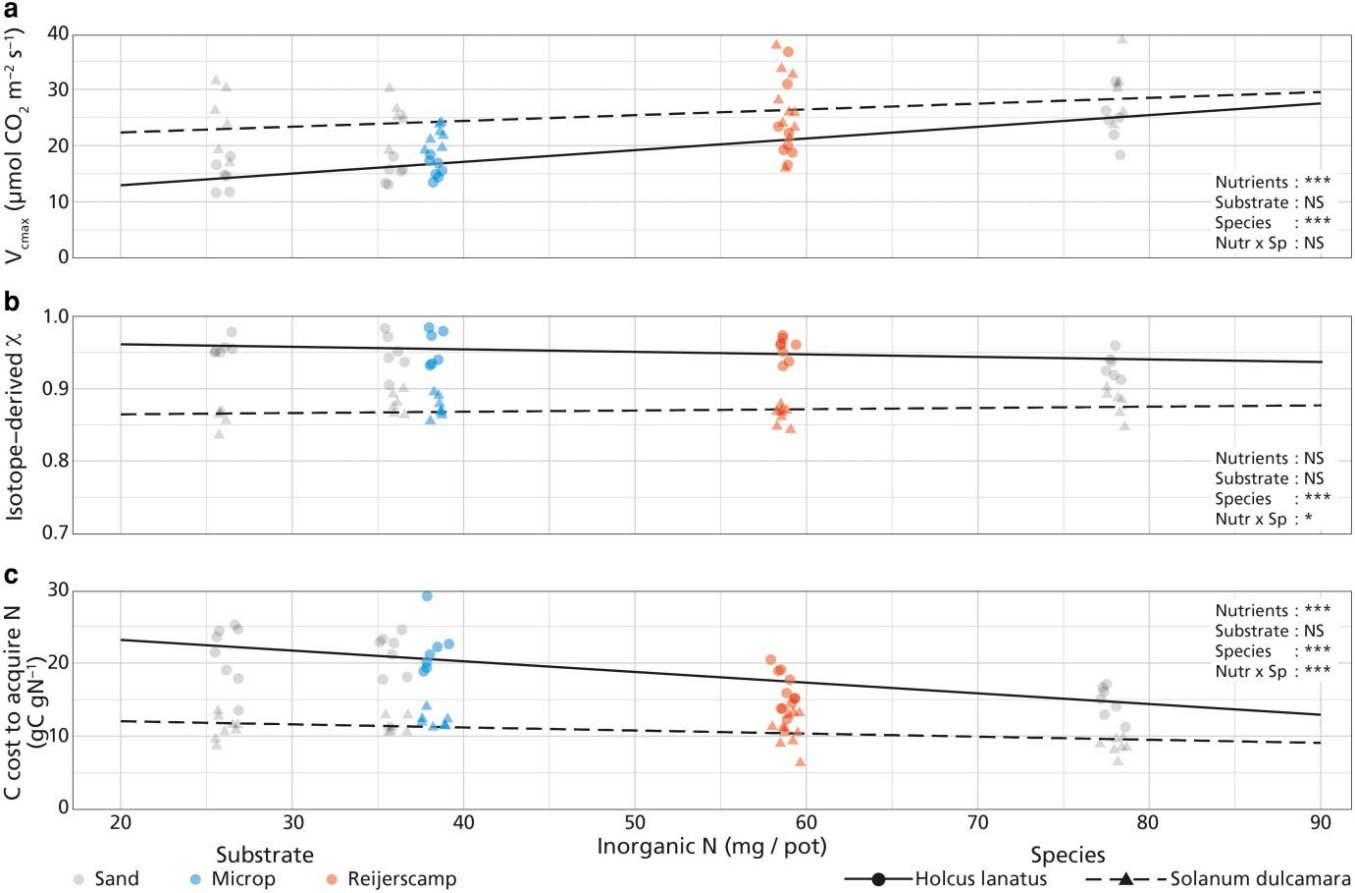
Response of *H. lanatus* (dots, solid line) and *S. dulcamara* (triangles, dashed lines) of a) maximum carboxylation capacity (*V*_cmax_, µmol m^−2^ s^−1^), b) isotope-derived *c_i_*/*c_a_* (*χ*_iso_) values (*y*-axis scaled to a representative range), and c) carbon cost to acquire nitrogen (gC gN^−1^) to nutrient content expressed in inorganic N per pot (*x*-axis). Data show the natural substrates using the measured nitrogen availability (filled shapes) and the total amount of nitrogen added to sand in the nutrient addition experiment run in the same year (open shapes). Trendlines were calculated using the ‘emtrends’ and ‘emmeans’ functions in the ‘emmeans’ R package (Lenth 2024). Significance determined using Type II *F*-tests by: *** = *P* < .001; ** = *P* < .01; * = *P* < .05, NS = *P* >0.05 (Table 4).

The different substrates (Sand, Microp, Reijerscamp) had no significant effect on *V*_cmax_ (Fig. 3a, *P* = .206, Table 4), *χ*_iso_ values (Fig. 3b, *P* = .646, Table 4), or carbon cost for nitrogen acquisition (Fig. 3c, *P* = .123, Table 4) when accounting for plant-available nitrogen. Both species significantly differed for all three variables, with *χ*_iso_ values and carbon cost for nitrogen acquisition higher in *H. lanatus* (*P* < .001, and *P* < .001, respectively, Table 4), and *V*_cmax_ higher in *S. dulcamara* (*P* < .001, Table 4). A significant interaction between nitrogen and species was detected for *χ*_iso_ (*P* = .043, Table 4) and carbon cost for nitrogen acquisition (*P* < .001, Table 4), indicating stronger responses to nutrient availability for *H. lanatus* than for *S. dulcamara*. There was no significant interaction between nutrient availability and species for *V*_cmax_ (*P* = .099, Table 4). These results highlight that nutrient availability drives differences in nitrogen acquisition costs and physiology in a slight species-specific manner, with overall limited substrate effects. Additional models regressing these traits against plant-available phosphorus show the same pattern (Supplementary Table SI3).

**Table 4.** ANOVA results summarizing the effects of nutrient amendment, substrate (sand, Microp, or Reijerscamp), species, and the interaction between nutrients and species for various plant traits.

Independent variable	*V*_cmax_	*χ*_iso_	Carbon cost for nitrogen acquisition
Nutrient availability (N)	**<0**.**001**	0.499	**<0**.**001**
Substrate	0.206	0.646	0.123
Species	**<0**.**001**	**<0**.**001**	**<0**.**001**
Nutrients:species	*0*.*099*	**0**.**043**	**<0**.**001**

*Only data of the nutrient addition experiment in 2023 is used, in addition to only the natural soils data of the same year. ‘Nutrient availability’ is treated as soil inorganic nitrogen. ‘Substrate’ is treated as categorical with three levels: Sand, Microp, and Reijerscamp. See Supplementary Table SI3 for the same analysis using ‘Nutrient availability’ as soil inorganic phosphorus. Significance determined using Type II *F*-tests (*α* = 0.05). *P*-values < .05 are indicated in bold.

## Discussion

In this experiment, we explored the link between leaf-level optimality and plant-level nitrogen acquisition costs across different soil environments. In support of our first hypothesis, we found a plant-level decrease in the carbon cost to acquire nitrogen with increasing nutrient addition. This response was driven by a larger increase in whole-plant nitrogen biomass than below-ground carbon biomass with increasing nutrient availability and was observed in both the nutrient addition experiment and the natural soil comparison. Similar patterns in resource allocation are observed in nutrient addition experiments and are reproduced in a select group of terrestrial biosphere models that explicitly represent costs for nutrient acquisition (Shi et al. 2016, Allen et al. 2020, Perkowski et al. 2021, 2025a). As light, temperature, vapor-pressure deficit, and water availability were similar between all treatments within a given experiment year, plant-level carbon cost to acquire nitrogen can be assumed to be driven by changes in nutrient supply and not demand for building and maintaining photosynthetic enzymes (Smith et al. 2019, Perkowski et al. 2021, Waring et al. 2023, Stocker et al. 2025). The between-year weather stochasticity and difference in run-time likely resulted in species-specific differences in plant growth, as suggested by the strong effect of year on whole-plant traits and the interactions between year and treatment. Consequently, whole-plant nitrogen biomass and root carbon biomass differed between the two iterations of the experiment. However, the structural carbon cost to acquire nitrogen did not, suggesting a strong nutrient control independent of variations in temperature, VPD, and run-time.

While root structural carbon provides an estimate of plant investment in nitrogen acquisition, it captures only part of the total carbon costs involved. In addition to the construction and maintenance of standing root biomass, plants incur carbon costs through other mechanisms involved in nitrogen acquisition, such as root exudation. For instance, the release of nonstructural carbohydrates into the rhizosphere can enhance nutrient availability via microbial or chemical processes (Carvalhais et al. 2011, Bengtson et al. 2012), representing a nonstructural carbon cost that plants can actively adjust in response to nutrient limitation (Dakora and Phillips 2002). Solely assessing structural root biomass as a proxy may therefore underestimate total carbon expenditure, particularly in soils that vary in biotic and abiotic constraints, which can impose differing respiratory or exudation demands on the plant (Cheng et al. 2014). These nonstructural costs are not necessarily proportional to root biomass but are likely to covary with structural investment. Additionally, the form of nitrogen available (nitrate vs. ammonium) can significantly alter the respiratory cost of uptake and assimilation (Bloom et al. 2002), contributing to variability not captured by structural investment alone. Nonetheless, root structural carbon cost remains a valuable indicator of plant nitrogen acquisition effort, especially in short-term pot experiments (Perkowski et al. 2021, 2025a). Additionally, our metric carbon cost to acquire nitrogen captures variation in belowground investment relative to plant nitrogen acquisition, but does not account for the parallel role of phosphorus, which increased by a similar proportion as nitrogen in our nutrient treatments from low to high supply and across natural soils. As a consequence, our cost metric is framed specifically around nitrogen, yet the observed patterns may also reflect broader nutrient-acquisition strategies involving phosphorus availability (Lu et al. 2022). Evidence from factorial N × P experiments shows that phosphorus supply interacts with nitrogen supply, influencing photosynthesis and biomass allocation (Güsewell 2004; Ghannoum and Conroy 2007; Wang et al. 2021b). While N and P uptake are fundamentally different processes (Muratore et al. 2021; Lambers 2022; Zhao et al. 2024), they can also be viewed as interdependent in terms of acquisition and assimilation costs (Vance et al. 2003; Ågren 2008). Linear regressions using soil available phosphorus instead of nitrogen revealed similar patterns, suggesting that our observed carbon–nitrogen tradeoffs reflect broader nutrient-acquisition strategies (Supplementary Table SI3).

Partially supporting our second hypothesis, both plants exhibited greater maximum rates of carboxylation with increasing nutrient availability. A common paradigm is that soil nitrogen must be in sufficient supply to increase *V*_cmax_, as photosynthetic enzymes such as RuBisCO require high nitrogen investments to build and maintain (Evans 1989, Evans and Seemann 1989, Zhu et al. 2019). Despite this, strong and consistent relationships between soil nitrogen supply and apparent photosynthetic capacity are not as commonly documented (Luo et al. 2021, Stocker et al. 2025), especially under high nutrient availability, where soil nutrient availability exceeds plant demand to build and maintain photosynthetic enzymes (Waring et al. 2023). This may be because plants increase nutrient allocation to leaf structural tissue or prioritize allocating nutrients towards creating a greater number of optimally coordinated leaves instead of overinvesting resources to photosynthetic enzymes in any given single leaf (Smith et al. 2019, Smith and Keenan 2020, Waring et al. 2023, Stocker et al. 2025, Perkowski et al. 2025a). In this experiment, we deliberately chose N and P levels to be linked to—and comparable with—inorganic nutrient availability in the compared natural soils, making the overall nutrient addition in comparison to other nutrient addition experiments relatively low. Thus, the positive effects of increasing nutrient availability on the maximum rate of Rubisco carboxylation were expected, as nutrient supply was likely insufficient to satisfy the demand for building photosynthetic enzymes. In this experiment, we observed a linear effect of nitrogen on *V*_cmax_, while linearity is likely not to be expected when nitrogen availability is increased *n*-fold (Tian et al. 2016, Perkowski et al. 2021, 2025a, Waring et al. 2023).

In contrast to our second hypothesis, we found no leaf-level decrease in *χ*_iso_ with increasing nutrient availability, supporting current EEO formulations. While current EEO frameworks attribute changes in *χ* to water supply and demand (Lavergne et al. 2020), they account for nitrogen use only through demand. More recently, changes in *χ* have been linked to edaphic influences irrespective of climate, but without mechanistic explanations (Paillassa et al. 2020, Westerband  et al. 2023, Cheaib et al. 2025). We hypothesized that increasing nutrient availability would reduce the plant-level carbon cost of nitrogen uptake and use relative to water (i.e. water becomes relatively more costly as nitrogen becomes less costly to acquire), and thus there would be a reduction in the leaf-level operational *c_i_*/*c_a_* ratio. The same acclimation response has been observed with decreased water availability, where plants shift investment towards photosynthetic capacity to maintain assimilation with reduced stomatal conductance during drought (Wright et al. 2001, Prentice et al. 2014, Xu et al. 2021). We did not observe lower *c_i_*/*c_a_* ratios through compensatory stomatal conductance reduction under nitrogen fertilization. Instead, plants increased their photosynthetic capacity and their stomatal conductance in tandem, making their relative investment in nitrogen use and water use similar, differing only in their magnitude. With nutrients likely constraining growth and photosynthesis in all treatments in our experiment, and water being ample available, plants may already have maximized daytime carbon uptake at the expense of water use efficiency. This strategy would maximize the nitrogen-use efficiency through maximizing stomatal conductance in all treatments and thus pushing *χ*_iso_ values near a species-specific physical maximum (Perkowski et al. 2025b). Observed *χ*_iso_ values in this experiment were at the upper end, or even exceeded previously reported *c_i_*/*c_a_* values for these species based on gas-exchange measurements. For example, *H. lanatus* has been reported to reach *c_i_*/*c_a_* values up to 0.9 (Gong et al. 2015), whereas in our study, values reached 0.98. Similarly, *S. dulcamara* has been reported up to ∼0.9 (Osmond 1983), which matches the highest values we observed here. Additionally, as the calculated leaf-lifespan *c_i_*/*c_a_* ratios through *χ*_iso_ (Eqs. 1 and 2) are amongst others a function of preferential fixation of the lighter carbon isotope ^12^C compared to the heavier ^13^C during photosynthetic CO_2_ fixation, this calculation has limitations. Both mesophyll conductance and post-photosynthetic fractionation might cause substantial variation in leaf carbon—water tradeoffs that are not captured currently but might also vary within and between species (de Boer et al. 2016, Ma et al. 2023). Additionally, carbon fixation in leaves takes place during the day, excluding potential nighttime processes affecting nitrogen- and water use efficiency, such as nighttime transpiration (Scholz et al. 2007, Wang et al. 2021a).

In the nutrient addition experiment (Figs 1 and 2, Tables 2 and 3), the main effects of nutrient supply on whole-plant nitrogen biomass, root carbon biomass, carbon cost for nitrogen acquisition, *V*_cmax_, and *N*_area_ were largely driven by significant changes in the High nutrient treatment, while pairwise comparisons showed no significant differences between Low and Medium treatments (Supplementary Tables SI5–SI7). This lack of response at the lower end likely reflects the unequal spacing of our nutrient treatments: medium had ∼1.4 times the availability of low, whereas high had ∼2.2 times the availability of Medium (Table 1). The design was chosen to reflect nutrient availabilities observed in natural soils, but the smaller contrast between the low and medium treatments limits the statistical interpretation of the pairwise tests. Nevertheless, regression analyses across the full nutrient gradient, including natural soils, met assumptions (homoscedasticity, normal residuals, no notable deviations) and consistently showed a linear decline in the carbon cost of nitrogen acquisition, a linear increase in *V*_cmax_, and no change for *χ*_iso_ with increasing nutrient availability (Fig. 3, Table 4).

We found that both plant- and leaf-level responses were primarily driven by species and soil nutrient availability, with no significant effects of substrate type. The lack of variation in photosynthetic traits or leaf physiological parameters between natural soils—beyond that explained by nutrient availability—suggests that naturally occurring soil microbiota exert minimal influence on the patterns predicted by the EEO framework. If soils imposed additional nutrient acquisition costs not reflected in, or disproportionate to, root structural carbon investment, we would expect corresponding changes in either *V*_cmax_ or *χ*_iso_ (Cheaib et al. 2025). Although ammonium-to-nitrate ratios differed only slightly across nutrient treatments and soil types (Table 1), these differences appear to have limited impact relative to absolute nitrogen availability, as no substrate effects were observed beyond those explained by total nitrogen, with the same patterns observed when regressed against phosphorus supply (Supplementary Table SI3). However, recent work suggests that the form of nitrogen may play an important role in dictating nitrogen-water use tradeoffs predicted from EEO theory (Perkowski et al. 2025b).

## Conclusion

Our results show that, although increasing nutrient availability decreased plant-level costs for nitrogen acquisition, the leaf-level ratio between water and nitrogen use did not change.

Plants allocated relatively less carbon below ground with more nitrogen return when nitrogen availability increases. This reduction in structural costs to acquire nitrogen was maintained when comparing plants in different natural soils with known quantities of nutrients, without any significant effect of soil substrate on measured photosynthetic traits. With an increase in nutrient availability, photosynthetic capacity also increased; similarly, within different substrates, a response that was likely driven by nutrient supply being insufficient for satisfying leaf-level demand for soil resources. Stable *χ*_iso_ values alongside increasing *V*_cmax_ imply that plants with higher photosynthetic capacity also exhibited higher stomatal conductance, likely facilitated by the high water availability. This lack of response of the leaf-level tradeoff between nitrogen and water to soil nutrient availability has been assumed in current EEO model formulations, though the absolute increase in *V*_cmax_ was not. Our findings thus reveal a decoupling between plant-level allocation costs and leaf-level optimality, suggesting that short-term (weeks to months) responses to nutrient availability may not significantly alter leaf-level economic tradeoffs. In contrast, previous studies reporting edaphic effects on *χ* (e.g. Paillassa et al. 2020, Westerband et al. 2023) may reflect longer-term acclimation or evolutionary adaptation.

It is important to note that we did not measure root microbial associations in this experiment. Symbioses with fungi or bacteria may represent a mechanism for plants to alter both plant-level and leaf-level costs simultaneously, potentially reducing nutrient acquisition costs without affecting water uptake (Cheaib et al. 2025). We therefore encourage further research into how microbial interactions and longer-term acclimation shape both the relative leaf-level tradeoffs and the absolute costs of resource acquisition. Such work will be essential to refine and expand the application of EEO theory in predictive modelling frameworks.

## Supplementary Material

plaf061_Supplementary_Data

## Data Availability

All data used for figures and data analysis are available on GitHub and Zenodo. https://github.com/JLankhorst/UU_NP_Soil. https://doi.org/10.5281/zenodo.17367398
