# Supplementary material for: Nutrient availability increases photosynthetic capacity without altering the cost of resource use for photosynthesis
Source: AoB Plants. 2025 Oct 22;17(6):plaf061. doi: 10.1093/aobpla/plaf061 (PMC12700163; doi:10.1093/aobpla/plaf061)
Supplement: plaf061_Supplementary_Data [file plaf061_supplementary_data.pdf]

Supporting Information

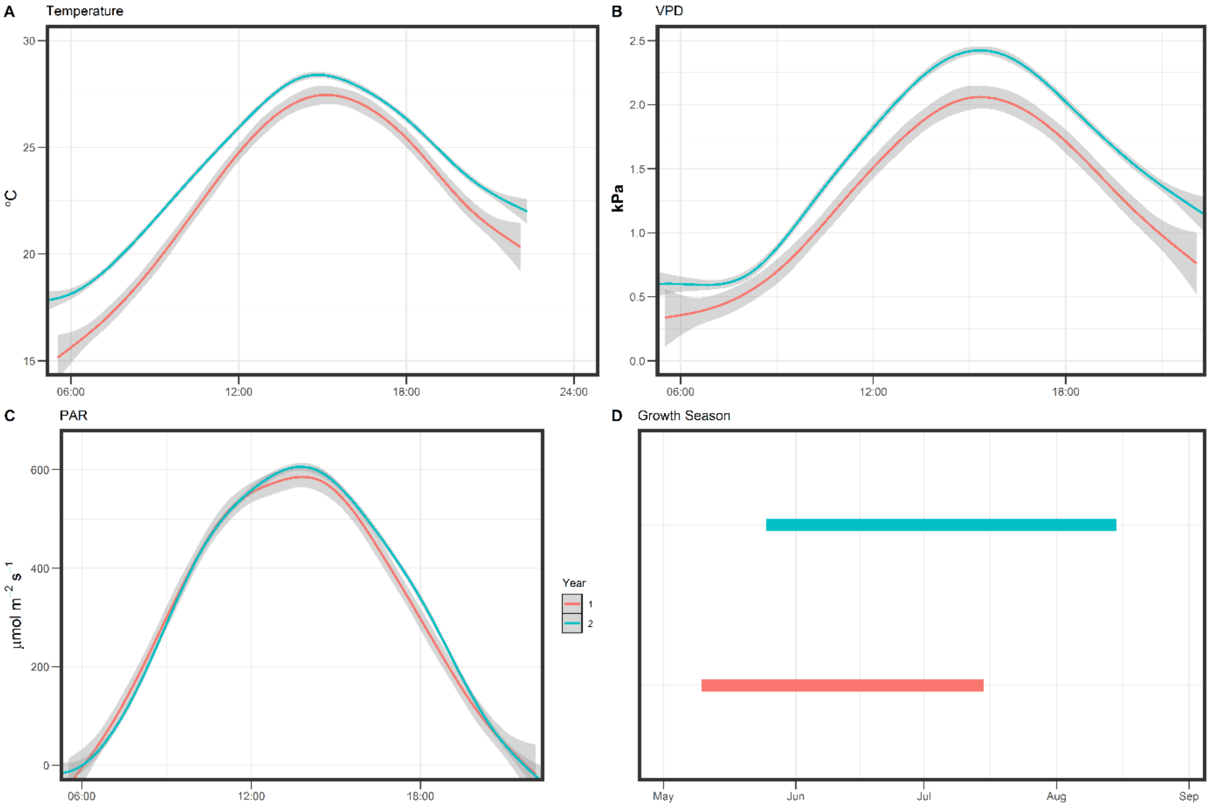

Figure SI1. **Growth conditions in the greenhouse during the two iterations of the experiment.** *A) Daytime temperatures (˚C), B) Daytime VPD (Kpa) C) Daytime PAR (µmol/m²/s) D) growth season. Plots A, B, and C were fitted with a non-parametric smoothing function and a 95% confidence interval.*

Table SI1. Averaged growth conditions during the two iterations of the experiment. Measured values in mean ± Std Dev.

Year	Experimental setup*	Mean daytime temperature (°C)	Mean nighttime temperature (°C)	Mean daytime PAR (µmol/m²/s)	Mean daytime max PAR (µmol/m²/s)	Mean daytime VPD (kPa)
2022	Sand (n=60):	22.00 ± 0.18	21.06 ± 0.18	472 ± 189	1165 ± 153	1.16 ± 0.70
2023	Sand (n=44): Reijerscamp (n=22) Microp (n=14)	24.65 ± 2.90	19.55 ± 1.78	464 ± 163	1101 ± 203	1.54 ± 0.73

**For a description of "Sand" see: Nutrient treatments. For a description of Reijerscamp and Microp, see: Natural soils.*

Table SI2. ANOVA results summarizing the effects of nutrient treatment, species, year, and the interactions between nutrient treatment and species, species and year, and nutrient treatment and year on the traits included in the manuscript.*

Trait	Whole-plant nitrogen biomass	Root carbon biomass	Carbon cost for nitrogen acquisition	V_{cmax}	LeafNarea	χ_{iso}
Independent variable						
Treatment	**<0.001**	**<0.001**	**<0.001**	**<0.001**	**<0.001**	0.404
Species	**<0.001**	**<0.001**	0.357	**<0.001**	**<0.001**	**<0.001**
Year	**<0.001**	**<0.001**	**<0.001**	0.123	**<0.001**	0.729
Treatment : Species	**<0.001**	0.426	**0.029**	**<0.001**	0.756	0.283
Species : Year	**<0.001**	**0.006**	0.555	**<0.001**	0.533	0.221
Treatment : Year	**<0.001**	0.637	0.726	0.251	**0.028**	0.957

* Only data of the nutrient addition experiment in 2022 and 2023 is used, without the natural soils data of 2023. Significance determined using Type II F-tests (α=0.05). P-values < 0.05 are indicated in bold and P-values between 0.05 and 0.100 are in italics.

Table SI3. ANOVA results summarizing the effects of phosphorus availability, substrate, species, and the interaction of phosphorus and species on the traits included in the manuscript.

Trait	V_{cmax}	χ_{iso}	Carbon cost for nitrogen acquisition
Independent variable			
Nutrient availability (P)	**<0.001**	0.505	**<0.001**
Substrate	**0.019**	0.564	**0.003**
Species	**<0.001**	**<0.001**	**<0.001**
Nutrients : Species	**0.033**	0.231	**<0.001**

* Only data of the nutrient addition experiment in 2023 is used, in addition to the natural soils data of the same year. “Nutrient availability” is treated as soil available phosphorus. “Substrate” is treated as categorical with three levels: Sand, Microp, Reijerscamp. Significance determined using Type II F-tests (α=0.05). P-values < 0.05 are indicated in bold and P-values between 0.05 and 0.100 are in italics.

Table SI4. Total amount of nutrients supplied per plant, and nutrient solution recipe for both iterations of the nutrient fertilization experiment in sand.

	Unit	Low	Medium	High
Element				
N	Mg/plant	26	36	78
P	Mg/plant	1.75	2.4	5.25
K	Mg/plant	66.67	66.67	66.67
Ca	Mg/plant	8.6	8.6	8.6
Mg	Mg/plant	1.95	1.95	1.95
S	Mg/plant	2.6	2.6	2.6
Fe	Mg/plant	0.68	0.68	0.68
Cl	Mg/plant	42.4	49.2	25.8
Compound				
KCl	mM	119.90	95.92	37.35
CaCl2	mM	26.82	26.82	26.82
MgSO4	mM	10.03	10.03	10.03
Fe-EDTA	mM	1.53	1.53	1.53
CuSO4	mM	0.01	0.01	0.01
H3BO3	mM	0.75	0.75	0.75
MnSO4	mM	0.08	0.08	0.08
Na2MoO4	mM	0.01	0.01	0.01
ZnSO4	mM	0.03	0.03	0.03
KNO3	mM	86.18	107.53	154.60
NH4NO3	mM	72.92	106.87	270.74
KH2PO4	mM	7.06	9.69	21.19

	Mean ± SE Narea / Mean ± SE Vcmax	**Low Y1 Holcus** 11.80 ± 1.62	**Medium Y1 Holcus** 17.00 ± 1.54	**High Y1 Holcus** 26.30 ± 1.62	**Low Y2 Holcus** 13.50 ± 1.58	**Medium Y2 Holcus** 18.70 ± 1.54	**High Y2 Holcus** 28.00 ± 1.58	**Low Y1 Solanum** 21.60 ± 1.62	**Medium Y1 Solanum** 21.90 ± 1.62	**High Y1 Solanum** 25.10 ± 1.54	**Low Y2 Solanum** 23.30 ± 1.58	**Medium Y2 Solanum** 23.60 ± 1.58	**High Y2 Solanum** 26.70 ± 1.54
**Low Y1 Holcus**	**0.220 ± 0.01190**		−5.162 ± 2.06 (n.s.)	−14.485 ± 2.10 (***)	−1.689 ± 1.20 (n.s.)	−6.851 ± 2.41 (n.s.)	−16.174 ± 2.42 (***)	−9.801 ± 2.10 (**)	−10.095 ± 2.10 (***)	−13.250 ± 2.06 (***)	−11.489 ± 2.42 (***)	−11.784 ± 2.42 (***)	−14.939 ± 2.41 (***)
**Medium Y1 Holcus**	**0.247 ± 0.01210**	+0.02766 ± 0.01590 (n.s.)		−9.323 ± 2.06 (**)	+3.473 ± 2.35 (n.s.)	−1.689 ± 1.20 (n.s.)	−11.012 ± 2.35 (***)	−4.638 ± 2.06 (n.s.)	−4.933 ± 2.06 (n.s.)	−8.088 ± 2.01 (**)	−6.327 ± 2.35 (n.s.)	−6.622 ± 2.35 (n.s.)	−9.777 ± 2.34 (**)
**High Y1 Holcus**	**0.312 ± 0.01210**	+0.09264 ± 0.01590 (***)	+0.06498 ± 0.01614 (**)		+1.689 ± 1.20 (n.s.)	+7.634 ± 2.41 (n.s.)	+1.235 ± 2.06 (n.s.)	+4.685 ± 2.10 (n.s.)	+4.390 ± 2.10 (n.s.)	+1.235 ± 2.06 (n.s.)	+2.996 ± 2.42 (n.s.)	+2.701 ± 2.42 (n.s.)	−0.454 ± 2.41 (n.s.)
**Low Y2 Holcus**	**0.272 ± 0.01210**	+0.05197 ± 0.00924 (***)	+0.02431 ± 0.01824 (n.s.)	−0.04066 ± 0.01824 (n.s.)		−5.162 ± 2.06 (n.s.)	−14.485 ± 2.10 (***)	−8.112 ± 2.42 (n.s.)	−8.406 ± 2.42 (*)	−11.561 ± 2.35 (***)	−0.000 ± 0.00	−9.801 ± 2.10 (**)	−13.250 ± 2.06 (***)
**Medium Y2 Holcus**	**0.299 ± 0.01250**	+0.07963 ± 0.01854 (**)	+0.05197 ± 0.00924 (***)	−0.01301 ± 0.01860 (n.s.)	+0.02766 ± 0.01590 (n.s.)		−9.323 ± 2.06 (**)	−6.327 ± 2.35 (n.s.)	−6.622 ± 2.35 (n.s.)	−8.088 ± 2.01 (**)	−4.638 ± 2.06 (n.s.)	−4.933 ± 2.06 (n.s.)	−8.088 ± 2.01 (**)
**High Y2 Holcus**	**0.364 ± 0.01250**	+0.14461 ± 0.01854 (***)	+0.11695 ± 0.01860 (***)	+0.05197 ± 0.00924 (***)	+0.09264 ± 0.01590 (***)	+0.06498 ± 0.01614 (**)		+6.373 ± 2.42 (n.s.)	+6.079 ± 2.42 (n.s.)	+2.924 ± 2.35 (n.s.)	+4.685 ± 2.10 (n.s.)	+4.390 ± 2.10 (n.s.)	+1.235 ± 2.06 (n.s.)
**Low Y1 Solanum**	**0.303 ± 0.01150**	+0.08333 ± 0.01544 (***)	+0.05567 ± 0.01569 (*)	-0.0931 ± 0.01596 (n.s.)	+0.03136 ± 0.01787 (n.s.)	+0.00370 ± 0.01823 (n.s.)	-0.06128 ± 0.01614 (*)		−0.295 ± 2.10 (n.s.)	−3.450 ± 2.06 (n.s.)	−1.689 ± 1.20 (n.s.)	−1.984 ± 2.42 (n.s.)	−5.139 ± 2.41 (n.s.)
**Medium Y1 Solanum**	**0.316 ± 0.01210**	+0.09612 ± 0.01590 (***)	+0.06846 ± 0.01614 (**)	+0.00348 ± 0.01614 (n.s.)	+0.04414 ± 0.01824 (n.s.)	+0.01649 ± 0.01860 (n.s.)	+0.04849 ± 0.01860 (n.s.)	+0.01279 ± 0.01569 (n.s.)		−3.155 ± 2.06 (n.s.)	−1.394 ± 2.42 (n.s.)	−1.689 ± 1.20 (n.s.)	−4.844 ± 2.41 (n.s.)
**High Y1 Solanum**	**0.384 ± 0.01210**	+0.16370 ± 0.01590 (***)	+0.13604 ± 0.01614 (***)	+0.07106 ± 0.01614 (**)	+0.11173 ± 0.01824 (***)	+0.08407 ± 0.01860 (**)	+0.01909 ± 0.01860 (n.s.)	+0.08037 ± 0.01569 (***)	+0.06758 ± 0.01614 (**)		+1.761 ± 2.35 (n.s.)	+1.466 ± 2.35 (n.s.)	−1.689 ± 1.20 (n.s.)
**Low Y2 Solanum**	**0.355 ± 0.01190**	+0.13530 ± 0.01812 (***)	+0.10764 ± 0.01817 (***)	+0.04266 ± 0.01817 (n.s.)	+0.08333 ± 0.01544 (***)	+0.05567 ± 0.01569 (*)	-0.00931 ± 0.01569 (n.s.)	+0.05197 ± 0.00924 (***)	+0.03918 ± 0.01817 (n.s.)	−0.02840 ± 0.01817 (n.s.)		−0.295 ± 2.10 (n.s.)	−3.450 ± 2.06 (n.s.)
**Medium Y2 Solanum**	**0.368 ± 0.01250**	+0.14809 ± 0.01854 (***)	+0.12043 ± 0.01860 (***)	+0.05545 ± 0.01860 (n.s.)	+0.09612 ± 0.01590 (***)	+0.06846 ± 0.01614 (**)	+0.00348 ± 0.01614 (n.s.)	+0.06476 ± 0.01823 (*)	+0.05197 ± 0.00924 (***)	−0.01561 ± 0.01860 (n.s.)	+0.01279 ± 0.01569 (n.s.)		−3.155 ± 2.06 (n.s.)
**High Y2 Solanum**	**0.435 ± 0.01250**	+0.21567 ± 0.01854 (***)	+0.18801 ± 0.01860 (***)	+0.12304 ± 0.01860 (***)	+0.16370 ± 0.01590 (***)	+0.13604 ± 0.01614 (***)	+0.07106 ± 0.01614 (**)	+0.13234 ± 0.01823 (***)	+0.11956 ± 0.01860 (***)	+0.05197 ± 0.00924 (***)	+0.08037 ± 0.01569 (***)	+0.06758 ± 0.01614 (**)	

Table SI5. Means ± Standard Errors and Tukey-adjusted pairwise comparisons of V_{cmax} (µmol m^{-2} s^{-1}) and N_{area} (g m^{-2}) of all combinations of species by treatment by year of the nutrient addition experiment. The significance of the pairwise comparisons between groups were calculated and adjusted for multiple comparisons using the Tukey HSD method. Each cell in the matrix shows the difference (row – column) between the two groups as: mean difference ± Standard Errors, significance (*** if $p < 0.001$, ** if $p < 0.01$, * if $p < 0.05$, n.s if $p > 0.05$)

		**Low Y1 Holcus**	**Medium Y1 Holcus**	**High Y1 Holcus**	**Low Y2 Holcus**	**Medium Y2 Holcus**	**High Y2 Holcus**	**Low Y1 Solanum**	**Medium Y1 Solanum**	**High Y1 Solanum**	**Low Y2 Solanum**	**Medium Y2 Solanum**	**High Y2 Solanum**
	**Mean ± SE Root carbon**	**9.85±1.35**	**16.02±1.38**	**42.01±1.38**	**39.66±1.38**	**45.84±1.43**	**71.82±1.43**	**11.05±1.31**	**19.89±1.38**	**53.12±1.38**	**40.87±1.36**	**49.70±1.43**	**82.94±1.43**
	**Mean ± SE whole-plant nitrogen**												
**Low Y1 Holcus**	**297 ± 28.0**		−6.17 ± 1.81 (*)	−32.16 ± 1.81 (***)	−29.82 ± 1.05 (***)	−35.99 ± 2.11 (***)	−61.97 ± 2.11 (***)	−1.21 ± 1.76 (n.s.)	−10.04 ± 1.81 (***)	−43.27 ± 1.81 (***)	−31.02 ± 2.06 (***)	−39.85 ± 2.11 (***)	−73.09 ± 2.11 (***)
**Medium Y1 Holcus**	**430 ± 28.5**	+133.1 ± 37.4 (n.s.)		−25.98 ± 1.83 (***)	−23.64 ± 2.07 (***)	−29.82 ± 1.05 (***)	−55.80 ± 2.11 (***)	+4.97 ± 1.78 (n.s.)	−3.86 ± 1.83 (n.s.)	−37.10 ± 1.83 (***)	−24.85 ± 2.07 (***)	−33.68 ± 2.11 (***)	−66.91 ± 2.11 (***)
**High Y1 Holcus**	**624 ± 28.5**	+326.8 ± 37.4 (***)	+193.8 ± 38.0 (**)		+2.34 ± 2.07 (n.s.)	−3.83 ± 2.11 (n.s.)	−11.11 ± 1.83 (***)	+30.95 ± 1.78 (***)	+22.12 ± 1.83 (***)	−11.11 ± 1.83 (***)	+1.14 ± 2.07 (n.s.)	−7.70 ± 2.11 (*)	−40.93 ± 2.11 (***)
**Low Y2 Holcus**	**717 ± 28.5**	+419.8 ± 21.7 (***)	+286.7 ± 42.9 (***)	−92.9 ± 42.9 (n.s.)		−6.17 ± 1.81 (*)	−32.16 ± 1.81 (***)	+28.61 ± 2.03 (***)	+19.78 ± 2.07 (***)	−13.46 ± 2.07 (***)	−1.21 ± 1.76 (n.s.)	−10.04 ± 1.81 (***)	−43.27 ± 1.81 (***)
**Medium Y2 Holcus**	**850 ± 29.5**	+552.9 ± 43.6 (***)	+419.8 ± 21.7 (***)	+226.0 ± 43.8 (***)	+133.1 ± 37.4 (n.s.)		−25.98 ± 1.83 (***)	+34.78 ± 2.07 (***)	+25.95 ± 2.11 (***)	−7.28 ± 2.11 (*)	+4.97 ± 1.78 (n.s.)	−3.86 ± 1.83 (n.s.)	−37.10 ± 1.83 (***)
**High Y2 Holcus**	**1044 ± 29.5**	+746.6 ± 43.6 (***)	+613.5 ± 43.8 (***)	+419.8 ± 21.7 (***)	+326.8 ± 37.4 (***)	+193.8 ± 38.0 (**)		+60.77 ± 2.07 (***)	+51.93 ± 2.11 (***)	+18.70 ± 2.11 (***)	+30.95 ± 1.78 (***)	+22.12 ± 1.83 (***)	−11.11 ± 1.83 (***)
**Low Y1 Solanum**	**88 ± 27.1**	−209.5 ± 36.3 (***)	−342.6 ± 36.9 (***)	−536.3 ± 36.9 (***)	−629.2 ± 42.0 (***)	−762.3 ± 42.9 (***)	−956.1 ± 42.9 (***)		−8.83 ± 1.78 (***)	−42.07 ± 1.78 (***)	−29.82 ± 1.05 (***)	−38.65 ± 2.07 (***)	−71.88 ± 2.07 (***)
**Medium Y1 Solanum**	**170 ± 28.5**	−127.1 ± 37.4 (*)	−260.2 ± 38.0 (***)	−453.9 ± 38.0 (***)	−546.8 ± 42.9 (***)	−679.9 ± 43.8 (***)	−873.7 ± 43.8 (***)	+13.3 ± 9.1 (n.s.)		−33.23 ± 1.83 (***)	−20.98 ± 2.07 (***)	−29.82 ± 1.05 (***)	−63.05 ± 2.11 (***)
**High Y1 Solanum**	**355 ± 28.5**	+57.5 ± 37.4 (n.s.)	−75.6 ± 38.0 (n.s.)	−269.3 ± 38.0 (***)	−362.3 ± 42.9 (***)	−495.3 ± 43.8 (***)	−689.1 ± 43.8 (***)	+267.0 ± 36.9 (***)	+184.6 ± 38.0 (**)		+12.25 ± 2.07 (***)	+3.42 ± 2.11 (n.s.)	−29.82 ± 1.05 (***)
**Low Y2 Solanum**	**508 ± 28.0**	+210.3 ± 42.6 (**)	+77.2 ± 42.7 (n.s.)	−116.5 ± 42.7 (n.s.)	−209.5 ± 36.3 (***)	−342.6 ± 36.9 (***)	−536.3 ± 36.9 (***)	+419.8 ± 21.7 (***)	+337.4 ± 42.7 (***)	+152.8 ± 42.7 (*)		−8.83 ± 1.78 (***)	−71.88 ± 2.07 (***)
**Medium Y2 Solanum**	**590 ± 29.5**	+292.7 ± 43.6 (***)	+159.6 ± 43.8 (**)	−34.1 ± 43.8 (n.s.)	−127.1 ± 37.4 (*)	−260.2 ± 38.0 (***)	−453.9 ± 38.0 (***)	+502.2 ± 42.9 (***)	+419.8 ± 21.7 (***)	+235.2 ± 43.8 (***)	+13.3 ± 9.1 (n.s.)		−42.07 ± 1.78 (***)
**High Y2 Solanum**	**775 ± 29.5**	+477.3 ± 43.6 (***)	+344.2 ± 43.8 (***)	+150.5 ± 43.8 (*)	−362.3 ± 43.6 (***)	−495.3 ± 43.8 (***)	−689.1 ± 43.8 (***)	+686.8 ± 42.9 (***)	+604.4 ± 43.8 (***)	+419.8 ± 21.7 (***)	+267.0 ± 36.9 (***)	+184.6 ± 38.0 (**)	

Table SI6. Means ± Standard Errors and Tukey-adjusted pairwise comparisons of whole-plant nitrogen biomass (mg) and root carbon biomass (mg) of all combinations of species by treatment by year of the nutrient addition experiment. The significance of the pairwise comparisons between groups were calculated and adjusted for multiple comparisons using the Tukey HSD method. Each cell in the matrix shows the difference (row – column) between the two groups as: mean difference ± Standard Errors, significance (*** if $p < 0.001$, ** if $p < 0.01$, * if $p < 0.05$, n.s if $p > 0.05$)

			Low Y1 Holcus	Medium Y1 Holcus	High Y1 Holcus	Low Y2 Holcus	Medium Y2 Holcus	High Y2 Holcus	Low Y1 Solanum	Medium Y1 Solanum	High Y1 Solanum	Low Y2 Solanum	Medium Y2 Solanum	High Y2 Solanum
		**Mean ± SE Chi**	**0.944 ± 0.00645**	**0.944 ± 0.00618**	**0.934 ± 0.00675**	**0.946 ± 0.00660**	**0.946 ± 0.00648**	**0.936 ± 0.00675**	**0.865 ± 0.00660**	**0.878 ± 0.00709**	**0.874 ± 0.00709**	**0.867 ± 0.00692**	**0.880 ± 0.00693**	**0.875 ± 0.00693**
	**Mean ± SE C cost for N acquisition**													
**Low Y1 Holcus**	**20.02 ± 0.72**			−0.000 ± 0.008 (n.s.)	+0.010 ± 0.009 (n.s.)	−0.002 ± 0.005 (n.s.)	−0.002 ± 0.010 (n.s.)	+0.008 ± 0.009 (n.s.)	+0.079 ± 0.009 ***	+0.066 ± 0.009 ***	+0.070 ± 0.009 ***	+0.077 ± 0.010 ***	+0.064 ± 0.010 ***	+0.080 ± 0.009 ***
**Medium Y1 Holcus**	**20.75 ± 0.73**		+0.73 ± 0.96 (n.s.)		+0.010 ± 0.009 (n.s.)	−0.002 ± 0.010 (n.s.)	−0.002 ± 0.005 (n.s.)	+0.009 ± 0.010 (n.s.)	+0.079 ± 0.009 ***	+0.066 ± 0.009 ***	+0.070 ± 0.009 ***	+0.077 ± 0.010 ***	+0.064 ± 0.010 ***	+0.080 ± 0.009 ***
**High Y1 Holcus**	**14.60 ± 0.73**		−5.43 ± 0.96 (***)	−6.15 ± 0.98 (***)		−0.012 ± 0.010 (n.s.)	−0.012 ± 0.010 (n.s.)	−0.002 ± 0.005 (n.s.)	+0.060 ± 0.009 ***	+0.056 ± 0.009 ***	+0.061 ± 0.009 ***	+0.067 ± 0.010 ***	+0.054 ± 0.010 ***	+0.059 ± 0.009 ***
**Low Y2 Holcus**	**20.55 ± 0.73**		+0.52 ± 0.56 (n.s.)	−0.20 ± 1.10 (n.s.)	+0.52 ± 0.56 (n.s.)		−0.000 ± 0.008 (n.s.)	+0.010 ± 0.009 (n.s.)	+0.080 ± 0.009 ***	+0.067 ± 0.009 ***	+0.071 ± 0.009 ***	+0.078 ± 0.010 ***	+0.065 ± 0.010 ***	+0.081 ± 0.009 ***
**Medium Y2 Holcus**	**21.27 ± 0.76**		+1.25 ± 1.12 (n.s.)	+0.52 ± 0.56 (n.s.)	+6.68 ± 1.12 (***)	+0.73 ± 0.96 (n.s.)		+0.010 ± 0.009 (n.s.)	+0.080 ± 0.009 ***	+0.067 ± 0.009 ***	+0.071 ± 0.009 ***	+0.078 ± 0.010 ***	+0.065 ± 0.010 ***	+0.081 ± 0.009 ***
**High Y2 Holcus**	**15.12 ± 0.76**		+4.90 ± 1.12 (**)	+5.63 ± 1.12 (***)	+0.52 ± 0.56 (n.s.)	+5.43 ± 0.96 (***)	+6.15 ± 0.98 (***)		+0.070 ± 0.009 ***	+0.057 ± 0.009 ***	+0.061 ± 0.009 ***	+0.067 ± 0.010 ***	+0.054 ± 0.010 ***	+0.059 ± 0.009 ***
**Low Y1 Solanum**	**11.41 ± 0.70**		−8.61 ± 0.93 (***)	−9.34 ± 0.95 (***)	−3.19 ± 0.95 (*)	−9.14 ± 1.08 (***)	−9.86 ± 1.10 (***)	−3.71 ± 1.10 (*)		−0.013 ± 0.010 (n.s.)	−0.009 ± 0.010 (n.s.)	−0.007 ± 0.010 (n.s.)	−0.021 ± 0.010 (n.s.)	−0.005 ± 0.010 (n.s.)
**Medium Y1 Solanum**	**10.21 ± 0.73**		−9.82 ± 0.96 (***)	−10.55 ± 0.98 (***)	−4.39 ± 0.98 (**)	−10.34 ± 1.10 (***)	−11.07 ± 1.12 (***)	−4.92 ± 1.12 (**)	−1.21 ± 0.95 (n.s.)		+0.004 ± 0.010 (n.s.)	+0.006 ± 0.010 (n.s.)	−0.008 ± 0.010 (n.s.)	+0.008 ± 0.010 (n.s.)
**High Y1 Solanum**	**7.82 ± 0.73**		−12.20 ± 0.96 (***)	−12.93 ± 0.98 (***)	−6.77 ± 0.98 (***)	−12.72 ± 1.10 (***)	−13.45 ± 1.12 (***)	−7.30 ± 1.12 (***)	−3.59 ± 0.95 (***)	−2.38 ± 0.98 (n.s.)		+0.002 ± 0.010 (n.s.)	−0.012 ± 0.010 (n.s.)	+0.012 ± 0.010 (n.s.)
**Low Y2 Solanum**	**11.94 ± 0.73**		−8.09 ± 1.10 (***)	−8.81 ± 1.10 (***)	−2.66 ± 1.10 (n.s.)	−0.00 ± 0.00	−9.34 ± 0.95 (***)	−3.19 ± 0.95 (*)	+0.52 ± 0.56 (n.s.)	+1.73 ± 1.10 (n.s.)	+4.11 ± 1.10 (*)		−0.014 ± 0.010 (n.s.)	−0.001 ± 0.010 (n.s.)
**Medium Y2 Solanum**	**10.73 ± 0.76**		−9.30 ± 1.12 (***)	−10.02 ± 1.12 (***)	−3.87 ± 1.12 (*)	−9.82 ± 0.96 (***)	−10.55 ± 0.98 (***)	−4.39 ± 0.98 (**)	−0.69 ± 1.10 (n.s.)	+0.52 ± 0.56 (n.s.)	+2.90 ± 1.12 (n.s.)	−1.21 ± 0.95 (n.s.)		+0.013 ± 0.010 (n.s.)
**High Y2 Solanum**	**8.35 ± 0.76**		−11.68 ± 1.12 (***)	−12.40 ± 1.12 (***)	−6.25 ± 1.12 (***)	−12.20 ± 0.96 (***)	−12.93 ± 0.98 (***)	−6.77 ± 0.98 (***)	−3.07 ± 1.10 (n.s.)	−1.86 ± 1.12 (n.s.)	+0.52 ± 0.56 (n.s.)	−3.59 ± 0.95 (***)	−2.38 ± 0.98 (n.s.)	

Table SI7. Means ± Standard Errors and Tukey-adjusted pairwise comparisons of isotope-derived c_i/c_a (χ_{iso}) and carbon cost for nitrogen acquisition (C cost for N acquisition, gN gC^{-1}) of all combinations of species by treatment by year of the nutrient addition experiment. The significance of the pairwise comparisons between groups were calculated and adjusted for multiple comparisons using the Tukey HSD method. Each cell in the matrix shows the difference (row – column) between the two groups as: mean difference ± Standard Errors, significance (*** if p < 0.001, ** if p < 0.01, * if p < 0.05, n.s if p > 0.05)
